# Reduced neuronal cell death after experimental brain injury in mice lacking a functional alternative pathway of complement activation

**DOI:** 10.1186/1471-2202-7-55

**Published:** 2006-07-14

**Authors:** Iris Leinhase, V Michael Holers, Joshua M Thurman, Denise Harhausen, Oliver I Schmidt, Malte Pietzcker, Mohy E Taha, Daniel Rittirsch, Markus Huber-Lang, Wade R Smith, Peter A Ward, Philip F Stahel

**Affiliations:** 1 Department of Trauma and Reconstructive Surgery, Charité University Medical School, Campus Benjamin Franklin, 12200 Berlin, Germany; 2 Departments of Medicine and Immunology, University of Colorado Health Sciences Center, Denver, CO 80262, USA; 3 Department of Pathology, University of Michigan Medical School, Ann Arbor, MI 48109, USA; 4 Department of Traumatology, University of Ulm Medical School, 89075 Ulm, Germany; 5 Department of Orthopaedic Surgery, Denver Health Medical Center, University of Colorado School of Medicine, Denver, CO 80204, USA

## Abstract

**Background:**

Neuroprotective strategies for prevention of the neuropathological sequelae of traumatic brain injury (TBI) have largely failed in translation to clinical treatment. Thus, there is a substantial need for further understanding the molecular mechanisms and pathways which lead to secondary neuronal cell death in the injured brain. The intracerebral activation of the complement cascade was shown to mediate inflammation and tissue destruction after TBI. However, the exact pathways of complement activation involved in the induction of posttraumatic neurodegeneration have not yet been assessed. In the present study, we investigated the role of the alternative complement activation pathway in contributing to neuronal cell death, based on a standardized TBI model in mice with targeted deletion of the factor B gene (*fB*-/-), a "key" component required for activation of the alternative complement pathway.

**Results:**

After experimental TBI in wild-type (*fB*+/+) mice, there was a massive time-dependent systemic complement activation, as determined by enhanced C5a serum levels for up to 7 days. In contrast, the extent of systemic complement activation was significantly attenuated in *fB*-/- mice (*P *< 0.05,*fB*-/- *vs*. *fB*+/+; *t *= 4 h, 24 h, and 7 days after TBI). TUNEL histochemistry experiments revealed that posttraumatic neuronal cell death was clearly reduced for up to 7 days in the injured brain hemispheres of *fB*-/- mice, compared to *fB*+/+ littermates. Furthermore, a strong upregulation of the anti-apoptotic mediator Bcl-2 and downregulation of the pro-apoptotic Fas receptor was detected in brain homogenates of head-injured *fB*-/- vs. *fB*+/+ mice by Western blot analysis.

**Conclusion:**

The alternative pathway of complement activation appears to play a more crucial role in the pathophysiology of TBI than previously appreciated. This notion is based on the findings of (*a*) the significant attenuation of overall complement activation in head-injured *fB*-/- mice, as determined by a reduction of serum C5a concentrations to constitutive levels in normal mice, and (*b*) by a dramatic reduction of TUNEL-positive neurons in conjunction with an upregulation of Bcl-2 and downregulation of the Fas receptor in head-injured *fB*-/- mice, compared to *fB*+/+ littermates. Pharmacological targeting of the alternative complement pathway during the "time-window of opportunity" after TBI may represent a promising new strategy to be pursued in future studies.

## Background

The high incidence of adverse outcomes after traumatic brain injury (TBI) has been attributed in large part to secondary mechanisms of neuronal cell death [1,2]. These include the induction of neuronal apoptosis and complement-mediated neuronal cell lysis [3-7]. Recent evidence suggests that the intracerebral activation of the complement cascade influences the fate of neurons by other than just the "classical" neuroinflammation-mediated effects [8-10]. For example, neuronal apoptosis can be induced by complement activation products, e.g. by binding of the anaphylatoxin C5a to its receptor (C5aR/CD88) expressed on neurons [11-15]. In addition, complement-mediated neuronal cell lysis can occur through the membrane attack complex (MAC; C5b-9) following inactivation of the physiological cellular protection mechanisms against homologous complement-mediated cell death [16-20]. Insights from recent experimental studies on intracerebral MAC injection underline the important role of the membrane attack pathway of complement in contributing to secondary neurodegeneration [21,22]. Posttraumatic complement activation and tissue deposition of the MAC were furthermore demonstrated in injured human and rodent brains by immunohistochemistry [18,23-26]. In addition, we have reported elevated levels of soluble MAC in human cerebrospinal fluid (CSF) after severe head injury [27].

Up to date, most studies which investigated the role of complement activation in the injured brain have focused on the effects of the complement cascade at a point where all three activation pathways converge, i.e. at the level of C3 or further downstream in the cascade [26,28-33]. Thus, the role which the individual pathways of complement activation play in the pathophysiology of TBI has not yet been determined. Recent studies established the alternative pathway of complement activation as a "key player" in the pathogenesis of ischemia/reperfusion-mediated inflammatory diseases outside the CNS [34]. For example, complement activation in renal ischemia/reperfusion injury was shown to be mediated almost exclusively by the alternative pathway [34-36]. In clinical studies on TBI patients, we have reported elevated levels of the crucial components required for alternative pathway complement activation, factor B and C3, in the CSF of severely head-injured patients [37].

Here, we demonstrate for the first time an important role of the alternative complement pathway in contributing to posttraumatic neuronal cell death, based on a standardized TBI model in factor B gene-deficient mice.

## Results and discussion

### Complement activation is attenuated in brain-injured *fB*-/- mice

**Figure 1:**
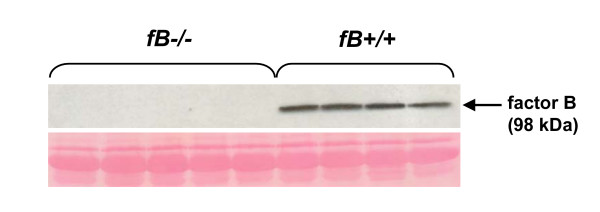
**Screening of serum samples from *fB*-/- and *fB*+/+ mice for factor B protein, as shown for an exemplary Western blot**. Samples were run out on SDS-PAGE, transferred to nitrocellulose membranes, and analyzed with a specific monoclonal anti-mouse factor B antibody and detection by chemiluminescence (ECL^® ^system, Amersham). A specific 98 kDa band, corresponding to murine fB, is detectable in the *fB*+/+, but not in the *fB*-/- mice. Each lane is representative of an individual mouse. The bottom panel shows equal protein loading in the different bands by ponceau red staining.

Screening of serum samples from all *fB*-/- mice and wild-type littermates (*fB*+/+) used in the present study revealed that factor B was only detectable in serum of *fB*+/+ animals, but not in the *fB*-/- mice. These control experiments were performed to ascertain that the knockout mice are completely devoid of factor B in serum. An exemplary Western blot is shown in Fig. 1.

**Figure 2:**
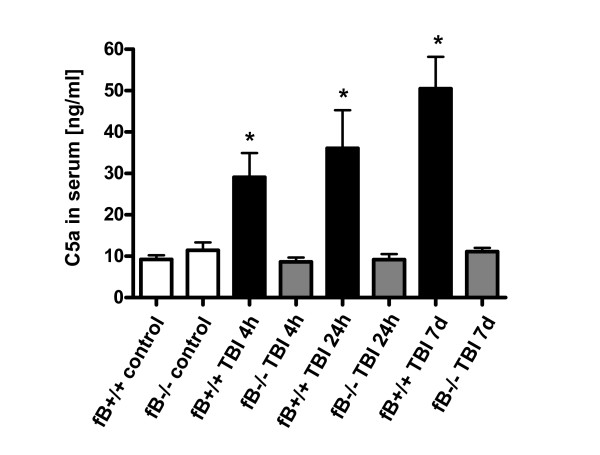
**Elevated C5a levels in serum of brain-injured C57BL/6 (*fB*+/+) mice are significantly attenuated in factor B gene-deficient (*fB*-/-) mice lacking a functional alternative complement pathway**. Serum samples from brain-injured *fB*+/+ and *fB*-/- mice of the C57BL/6 strain (*n *= 6 per group and time-point) and from normal C57BL/6 mice (control; *n *= 4) and normal *fB*-/- mice (*n *= 4) were analyzed by ELISA specific for mouse C5a. Data are shown as mean levels ± SD. *P < 0.05, *fB*+/+ *vs*. control and *fB*+/+ *vs*. *fB*-/- mice. TBI, traumatic brain injury.

Experimental closed head injury in wild-type C57BL/6 mice resulted in a systemic activation of the complement cascade, as determined by significantly elevated serum levels of the complement activation product C5a at all time-points assessed from 4 hours to 7 days (*P *< 0.05 *vs*. normal mouse serum, unpaired Student's *t*-test; Fig. 2). In contrast, anaphylatoxin C5a serum levels were not induced in *fB*-/- mice at all corresponding time-points after head trauma and found to be in a similar range to baseline levels in normal C57BL/6 or *fB*-/- mice (*P *< 0.05 *vs*. brain-injured *fB*+/+ mice, unpaired Student's *t*-test; Fig. 2). The data indicate the lack of C5a increase in sera of *fB*-/- mice. It seems likely that this is due to impaired generation of C5a via the alternative pathway of complement activation, although accelerated metabolic destruction of C5a in the absence of factor B cannot be ruled out. These findings imply that the alternative pathway is the source for complement activation after brain injury, a notion which was previously substantiated for other inflammatory diseases outside the CNS, such as rheumatoid arthritis, autoimmune nephritis, and ischemia/reperfusion injuries [34,38].

Experimental head injury induced mean anaphylatoxin C5a levels in serum of around 50 ng/ml within 7 days in wild-type mice (Fig. 2). Serum C5a levels described in the literature due to triggers for complement activation other than CNS trauma are in the range of 100 ng/ml, as described for patients with severe sepsis [39]. With regard to central nervous system inflammation, we have previously reported significantly elevated C5a levels in human CSF of patients with bacterial meningitis (mean levels around 75 ng/ml), as compared to patients with aseptic/viral meningitis [40]. C5a levels were not detectable in normal CSF of *n *= 66 healthy humans with a lower limit of sensitivity of the assay at 1.5 ng/ml [40]. Thus, the serum C5a levels detected in brain-injured C57BL/6 mice in the present study appear to reflect concentrations of clinical relevance, as far as data derived from an experimental mouse model can be extrapolated to human disease.

### The lack of factor B leads to reduced neuronal cell death after head injury

**Figure 3:**
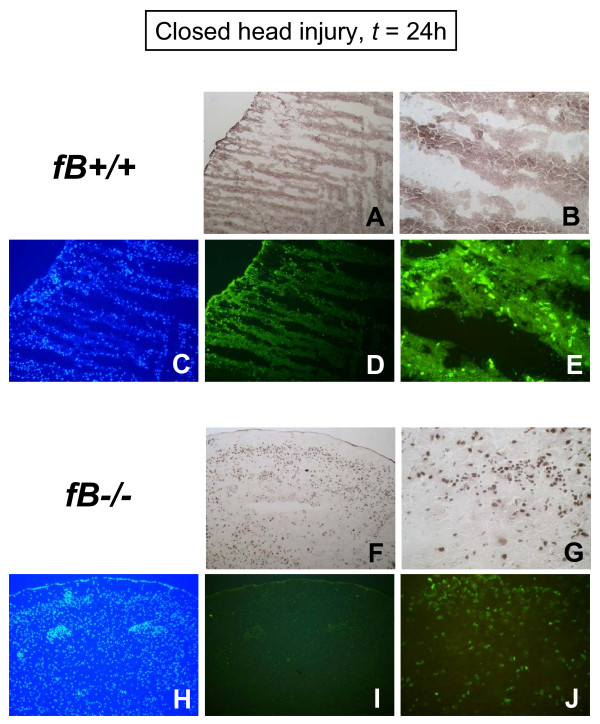
**Attenuated neuronal cell death in the injured hemisphere of factor B gene-deficient mice 24 hours after closed head injury**. Coronal cryosections of the left (injured) hemisphere of wild-type (*fB*+/+, **panels A-E**) and factor B knockout mice (*fB*-/-, **panels F-J**) were analyzed by immunohistochemistry with a specific antibody to the neuronal marker NeuN (**A, B, F, G**) or by TUNEL-histochemistry of adjacent sections (**D, E, I, J**). The overall cellular morphology of the TUNEL sections is revealed by DAPI nuclear stain (**C, H**). The panels **B, E, G, J **represent a 4-fold magnification of the respective panels **A, D, F, I**. The evident destruction of the cortical tissue in brain-injured wild-type mice (upper panels) appears to be structurally preserved in *fB*-/- mice (bottom panels). The data shown here are highly reproducible in all tissue sections and animals assessed. Original magnifications: 100× (**A, C, D, F, H, I**), 400× (**B, E, G, J**).

**Figure 4:**
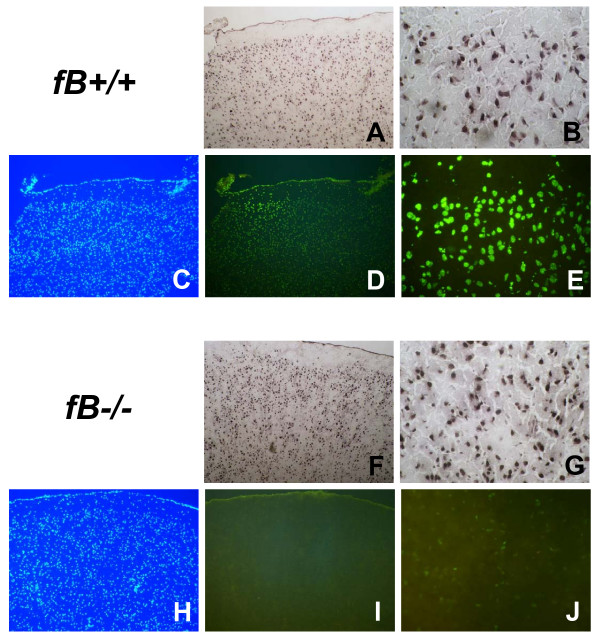
**Attenuated neuronal cell death in the injured hemisphere of factor B gene-deficient mice 7 days after closed head injury**. Coronal cryosections of the left (injured) hemisphere of wild-type (*fB*+/+, **panels A-E**) and factor B knockout mice (*fB*-/-, **panels F-J**) were analyzed by immunohistochemistry with a specific antibody to the neuronal marker NeuN (**A, B, F, G**) or by TUNEL-histochemistry of adjacent sections (**D, E, I, J**). The overall cellular morphology of the TUNEL sections is revealed by DAPI nuclear stain (**C, H**). The panels **B, E, G, J **represent a 4-fold magnification of the respective panels **A, D, F, I**. The data shown here are highly reproducible in all tissue sections and animals assessed. Original magnifications: 100× (**A, C, D, F, H, I**), 400× (**B, E, G, J**).

The mortality from brain injury in this model system is approximately 5–8% within 7 days, as previously reported [41]). There was no difference in mortality between *fB*-/- and *fB*+/+ mice. As previously described, neuronal cell death was observed in injured brains of wild-type C57BL/6 mice for up to 7 days after closed head injury [41]. An increase in TUNEL-positive cells was detected in the injured hemispheres of *fB*+/+ mice within 4 to 24 hours after trauma, persisting for up to 7 days (Fig. 3**and **Fig. 4; **panels D & E**; whereby **E **represents a 4-fold magnification of **D**). The nuclear staining with 4',6'-diamino-2-phenylindole (DAPI; **panel C**) shows the cellular morphology in adjacent sections to those assessed by TUNEL histochemistry. Neurons were determined as the main TUNEL-positive cell-type by immunohistochemical staining of adjacents sections with the specific cell-marker NeuN (Fig. 4, **panels A & B **whereby **B **represents a 4-fold magnification of **A**). The staining of astrocytes (anti-GFAP), microglia (anti-CD11b), and endothelial cells (anti-CD144) revealed that these resident cells in the brain are represent only a small part of TUNEL-positive cells, e.g. in the molecular layer of the cortex (Fig. 4). Furthermore, neurons were confirmed as the predominant TUNEL-positive cell-type by their typical cellular size and morphology (as opposed to glial cells) and to the typical neuronal layers of TUNEL-positive cells in the injured cortex (Fig. 4). The data shown in figures 3**and **4 are highly reproducible in all tissue sections and animals assessed and are representative for 2–3 mice of each group (*fB*+/+ and *fB*-/-) per time-point assessed. These findings corroborate previously published data on neuronal apoptosis in the current and other experimental TBI models as well as in head-injured patients [41-45]. In contrast to the extent of neuronal cell death in brain-injured *fB*+/+ mice, the *fB*-/- animals showed a clear reduction in TUNEL-positive neurons in injured brains from 4 hours to 7 days after trauma (Fig. 3**and **Fig. 4, **panels I & J ***vs*. **D & E**, respectively). In addition, the evident cortical tissue destruction in brain-injured wild-type mice at 24 hours (Fig. 3, upper panels) appears to be structurally preserved in *fB*-/- mice (Fig. 3, bottom panels). TUNEL-positive cells and extent of tissue destruction were less in the contralateral (right) hemisphere as compared to the injured (left) hemisphere at all time-points assessed, both in the knockout and the wild-type mice (data not shown). These findings support the recently established concept of complement-dependent regulation of neuronal apoptosis [7,10,15,46] and promote the *in vivo *significance of the alternative (factor B-dependent) pathway of complement activation in regulating the extent of secondary neurodegeneration after TBI. This is the first study, to our knowledge, which investigated exclusively the role of the alternative pathway in contributing to neuropathology after brain injury. The *fB*-/- mice have previously been shown to be protected from experimental demyelination in an animal model of multiple sclerosis [47]. The studies by Nataf and colleagues support our present findings in that the genetic deficiency of factor B, which provokes the complete lack of a functional alternative complement activation pathway, plays an essential role for neuroprotection in models of autoimmune and traumatic CNS injury.

### Upregulation of Bcl-2 and downregulation of Fas in injured *fB*-/- brains

**Figure 5:**
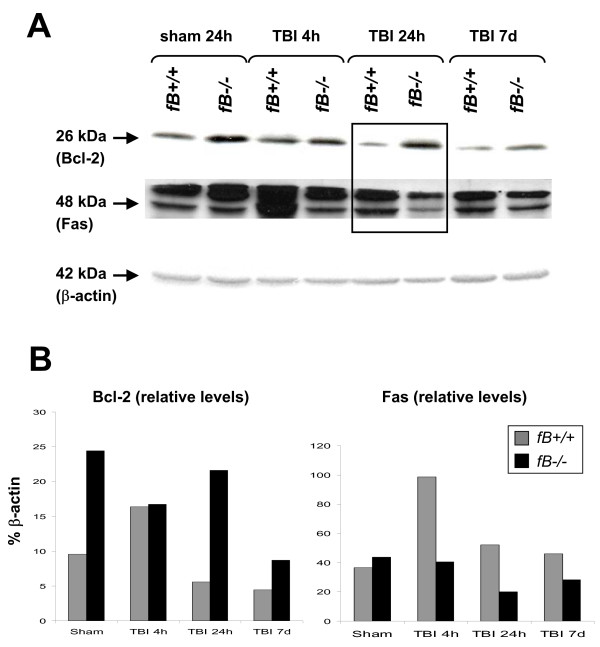
**Upregulation of Bcl-2 and downregulation of Fas receptor in brains of *fB*-/- mice after traumatic brain injury (TBI), as determined by Western blot analysis**. Homogenized brain tissue specimens from the injured hemispheres of sham-operated and head-injured *fB*+/+ and *fB*-/- mice were run out on SDS-PAGE, transferred to nitrocellulose membranes, and analyzed with specific monoclonal antibodies to Bcl-2, Fas, and β-actin as internal control and detection by chemiluminescence assay (ECL^® ^system, Amersham). (**A**) The visualized 26 kDa band corresponding to mouse Bcl-2 is enhanced in the knockout mice at 24 hours, compared to head-injured wild-type littermates. Furthermore, a downregulation in Fas receptor staining intensity is obvious in brain-injured knockout mice at all time-points assessed, compared to *fB*+/+ mice. The exemplary blot is representative of three independent experiments. (**B**) The individual band intensities of the blot shown in panel **A **were quantified by TINA 2.09 software (Raytest, Straubenhardt, Germany) and the data are shown as relative levels to the according β-actin band intensity.

Posttraumatic neuronal apoptosis has been shown to be promoted by the Fas-mediated extrinsic pathway and by a suppression of the mitochondrial anti-apoptotic mediator Bcl-2 of the intrinsic pathway of apoptosis [48-54]. By Western blot analysis, we found a marked upregulation of protective Bcl-2 protein levels in brain homogenates of sham-operated and head-injured *fB*-/- mice at 24 hours – and to a lesser degree at 7 days – after trauma, compared to *fB*+/+ littermates (Fig. 5). No differences in Bcl-2 expression between knockout and wild-type mice was seen at 4 h after TBI (Fig. 5). Interestingly, sham-operation alone was a sufficient trigger for inducing Bcl-2 protein expression in the brain of *fB*-/- mice within 24 h (Fig. 5). We have previously reported the phenomenon of increased intracerebral mediator expression by sham operation alone (i.e. anesthesia and scalp incision) such as for the anaphylatoxin C5a receptor (C5aR/CD88) in sham-operated C57BL/6 mice [13]. In the present study, it appears that the threshold for induction of Bcl-2 in the brain of sham-operated knockout mice is lower than in wild-type mice, thus possibly contributing to increased intracerebral Bcl-2 levels also in the brain-injured *fB*-/- mice. We plan to explore the pharmacological aspects of this phenomenon in future studies.

With regard to the extrinsic pathway of apoptosis, a marked downregulation in Fas receptor expression was seen within 4 hours to 7 days after TBI in *fB*-/- mice, compared to *fB*+/+ animals (Fig. 5). Although these data are not quantitative, the differences in staining intensity of the 26 kDa (Bcl-2) and 48 kDa bands (Fas) appear more intense in the brain-injured knockout mice than in the corresponding wild-type littermates at the above-mentioned time-points (Fig. 5). These findings suggest, but do not prove, an involvement of the alternative pathway of complement activation in regulating neuronal apoptosis after TBI by suppression of Bcl-2 and induction of Fas receptor expression in the injured brain. Both aspects are critical in the regulation of post-injury neuronal apoptosis, as previously determined by other investigators in different model systems [48-50]. An experimental study on a controlled cortical impact brain injury model demonstrated that the cortical lesion volume was significantly reduced in transgenic mice with over-expression of the Bcl-2 gene by 7 days after trauma, compared to wild-type littermates [55]. Thus, Bcl-2 was attributed an important role in the regulation of the mitochondrial (intrinsic) pathway of apoptosis after TBI [4,53,55,56].

We have recently shown that the pharmacological "pan"-inhibition of complement activation at the level of the C3 convertases by *Crry-Ig*, a murine recombinant chimeric fusion molecule, leads to enhanced intracerebral Bcl-2 gene and protein expression and to increased neuronal survival in the hippocampus of brain-injured mice [32]. Similar findings were described in a model of murine autoimmune cerebritis, where the blocking of complement activation by *Crry-Ig *resulted in a significant attenuation of neuronal apoptosis [15].

The data from the present study support the biological significance of the alternative pathway of complement activation in contributing to the neuropathological sequelae of TBI and provide the basis for future pharmacological studies with selective alternative pathway inhibitors, e.g. such as factor B antagonists [34,57].

## Conclusion

In summary, the present data provide first evidence of a major role of the alternative pathway of complement activation in contributing to the overall extent of posttraumatic complement activation (C5a generation) and to secondary neuronal cell death after brain injury (TUNEL, Bcl-2, and Fas data). This is a fairly new and provocative notion, since all previously published studies on experimental complement inhibition in TBI models have focussed on interfering with the complement cascade at the "common junction" level of C3 convertases [26,28-32] or further downstream in the cascade, e.g. by specific blocking of anaphylatoxin C5a or its receptor [30]. The hitherto underestimation of the pathophysiological role of the alternative complement pathway in the neuropathology of brain injury may be in part due to the historically established predominant role of the classical pathway in various neurological diseases [58,59]. However, the results from the present study imply that these insights may not necessarily reflect the "true" *in vivo *significance of the alternative complement pathway in a complex multifactorial neuroinflammatory disease, such as in the setting of TBI [60]. The fact that elevated factor B levels are present in the intrathecal compartment of severely head-injured patients [37] further supports the concept that the pharmacological targeting of factor B may present a reasonable basis for future investigations.

## Methods

### Factor B-/- mice

The genetic knockout mice deficient in factor B (*fB*-/-) were previously characterized and shown to have a complete lack of a functional alternative complement pathway [61]. They were originally created with Sv129 embryonic stem cells and crossed with C57BL/6 mice prior to expansion of the colony at F1. They were then back-crossed for more than 10 generations against a pure C57BL/6 background and found to be grossly indistinguishable from C57BL/6 mice [35]. Knockout mice and wild-type littermates (*fB*+/+) were acclimatized several weeks before the experiments and kept isolated from external influences during the entire time course of the study. They were bred in a selective pathogen-free (SPF) environment and standardized conditions of temperature (21°C), humidity (60%), light and dark cycles (12:12 h), with food and water provided *ad libitum*. Only male mice were used for this study in order to avoid a bias in gender with regard to levels of complement activity and to susceptibility to brain injury which seems to be significantly influenced by female reproductive hormones [62,63]. All experiments were performed in compliance with the standards of the *Federation of European Laboratory Animal Science Association *(FELASA) and were approved by the institutional animal care commitee (*Landesamt für Arbeitsschutz, Gesundheitsschutz und technische Sicherheit*, Berlin, Germany, No. G0099/03 and No. G0308/04).

### Brain injury model

Experimental closed head injury was performed in knockout (*fB*-/-) mice and wild-type littermates (*fB*+/+) of the C57BL/6 strain (*n *= 6 per group and time-point) using a standardized weight-drop device, as previously described [13,41,64-66]. In brief, after induction of isoflurane anesthesia, the skull was exposed by a midline longitudinal scalp incision. A 333 g weight was dropped on the fixed skull from a height of 2 cm, resulting in a focal blunt injury to the left hemisphere. After trauma, the mice received supporting oxygenation with 100% O_2 _until fully awake and were then brought back to their cages. At defined time-points (*t *= 4 h, 24 h, and 7 days), mice were euthanized and brain hemispheres were extracted for analysis by immunohistochemistry, TUNEL histochemistry, and SDS-PAGE/Western blot analysis. In addition, serum samples were collected for determination of complement anaphylatoxin C5a levels by ELISA and Western blot analysis of Bcl-2 (see below).

Sham-operated mice were kept under identical conditions as the trauma group and underwent the same procedures (anesthesia and scalp incision) except that no head injury was applied.

### ELISA for mouse C5a

For determination of complement anaphylatoxin C5a levels in serum samples of head-injured and normal C57BL/6 control mice, we used an ELISA developed in the laboratory of Dr. P.A. Ward (Ann Arbor, MI, USA), as previously described [67]. In brief, ELISA plates (Immulon 4HBX, Thermo Labsystems, Milford, MA, USA) were coated with purified monoclonal anti-mouse C5a IgG (5 μg/ml, BD Pharmingen, San Diego, CA, USA). This capture antibody recognizes both C5a and C5a-desArg, but not the precursor molecule C5. After blocking of non-specific binding sites with 1% milk (Roth, Karlsruhe, Germany) in PBS (Gibco-Invitrogen, Carlsbad, CA, USA) containing 0.05% TWEEN 20 (Sigma-Aldrich, St. Louis, MO, USA), the plate was coated with 100 μl serum diluted 1:20 (in 0.1% milk in PBS contaning 0.05% TWEEN) and murine recombinant mouse C5a at defined concentrations for establishing the standard curve. After incubation and subsequent washing steps, biotinylated monoclonal anti-mouse C5a antibody was added at 500 ng/ml (BD Pharmingen) followed by washing steps and incubation with streptavidin-peroxidase at 400 ng/ml (Sigma). For colorimetric reaction, the substrate (0.4 mg/ml OPD with 0,4 mg/ml urea hydrogen peroxide in 0.05 M phosphate citrate buffer; Sigma) was added and the color reaction was stopped with 3 M sulfuric acid. The absorbance was read at 490 nm ("SpectraMax 190" reader, Molecular Devices, Sunnyvale, CA, USA. All samples were analyzed in duplicate wells and results were calculated from the means of duplicate sample analysis. The standard curve was linear from 50 ng/ml to 0.1 ng/ml which represents the lower limit of detection of this assay.

### Western blot

All mice used in this study were screened by Western blot analysis for the presence of factor B in serum, as an internal quality control (Fig. 1). The protein levels of the mitochondrial anti-apoptotic mediator Bcl-2 and of the pro-apoptotic Fas receptor were determined in homogenized mouse brains and matched serum samples at 4 h, 24 h and 7d following head injury or sham operation in *fB*-/- and *fB*+/+ mice. The Western blot technique was previously described [32]. Briefly, mouse brains were extracted under anesthesia, separated into left and right hemispheres, and immediately homogenized in lysis buffer (Sigma) containing 100 mM TRIS-HCl (pH 7.5), 150 mM NaCl, 0.5% sodium dodecyl sulfate (SDS), 0.5% Nonidet P-40, 10 μg/ml aprotinin, 10 μg/ml leupeptin, 5 μg/ml pepstatin, 1 mM phenyl-methyl-sulfonyl fluoride in deionized water, using an Ultra Turrax Homogenizer^® ^(IKA Werke, Staufen, Germany). After 15 min centrifugation at 13,000 × g, the protein content of the supernatants was determined by commercially available colorimetric protein assay ("BCA Protein Assay", Pierce/Perbio Science, Bonn, Germany). A 60 μg sample of total protein was denatured in loading buffer and separated under reducing conditions on 12% SDS-polyacrylamide gels in parallel with a broad range prestained SDS-PAGE protein standard (Bio-Rad, Munich, Germany). Proteins were then transferred to Protran BA 83 nitrocellulose membranes (Schleicher & Schuell, Dassel, Germany) by electroblotting (Bio-Rad). Equal transfer of protein to the blotting membrane was confirmed by ponceau red staining (Sigma). The blots were blocked overnight and then incubated with either monoclonal anti-mouse Bcl-2 (Santa Cruz Biotechnology, Heidelberg, Germany), diluted 1:500, polyclonal rabbit anti-mouse Fas (clone A-20, Santa Cruz), diluted 1:200, polyclonal chicken anti-mouse anti-factor B, diluted 1:8,000 (kindly provided by Dr. Scott R. Barnum, University of Alabama at Birmingham, AL, USA) as primary antibodies, and with a monocloncal anti-β-actin antibody (clone AC-15, Sigma) diluted 1:10,000, as internal control for ascertaining equal loading of the bands. After incubation with peroxidase-labelled secondary antibodies (Dako, Hamburg, Germany, and Santa Cruz Biotechnology, Heidelberg, Germany), diluted 1:5,000, antibody binding was visualized by a non-radioactive chemiluminescence technique using a commercially available ECL^® ^Western blotting kit (Amersham Pharmacia Biotech, Freiburg, Germany). A semi-quantitative analysis of the individual band intensities was performed by scanning the films (hp Scanjet 5530, Hewlett-Packard, Böblingen, Germany) followed by quantification using the TINA 2.09 software (Raytest, Straubenhardt, Germany). The data are presented in histograms as relative levels to the according β-actin band intensity.

### Immunohistochemistry

For assessment of neuronal morphology, integrity, and apoptosis, extracted mouse brains were snap-frozen in liquid nitrogen, embedded in OCT compound (Sakura Finetek, Torrance, CA) and stored at -80°C until used for analysis. Six to eight-micrometer thick coronal tissue sections were cut with a cryostat at -20°C. For immunohistochemistry, slides were fixed in acetone and then analyzed by a standard biotin/avidin/peroxidase technique with DAB-tetrahydrochloride as chromogen (Vector, Burlingame, CA), as previously described [13,32]. The following primary antibodies were used as cell-markers: monoclonal anti-NeuN, at a titrated dilution of 1:2,000 (Chemicon, Hampshire, UK) for neurons; polyclonal rabbit anti-GFAP, 1:100 (Shandon Immunon, Pittsburgh, PA, USA) for astrocytes; monoclonal rat anti-CD11b, 1:100, (Accurate Chemical, Westbury, NY, USA) for microglia; polyclonal goat-anti CD144, 1:200 (Santa Cruz) for endothelial cells. Non-immunized IgG (Vector) was used as negative control at equal dilutions as the omitted specific antibody.

To determine the extent of intracerebral neuronal cell death, TUNEL histochemistry was performed using a "Fluorescein *In Situ *Cell Death Detection Kit" (Roche Diagnostics GmbH, Mannheim, Germany), according to the manufacturer's instructions, as previously described [41]. Briefly, slides were dried for 30 min followed by fixation in 10% formalin solution at RT. After washing in PBS (three times for 3 min), sections were incubated in ice-cold ethanol-acetic acid solution (2:1) for 5 min at -20°C. Thereafter, they were washed in PBS and incubated in a permeabilization solution with 3% Triton X-100 in PBS for 60 min at RT, then incubated with the TdT enzyme in a reaction buffer containing fluorescein-dUTP for 90 min at 37°C. Negative control was performed using only the reaction buffer without TdT enzyme. Positive controls were performed by digesting equal brain sections with DNase grade I solution (500 U/ml; Roche) for 20 min at RT and always kept separate from the other samples thereafter. After labelling, the sections were washed again in PBS and to visualize the unstained (TUNEL-negative) cells, the sections were covered with Vectashield^® ^mounting medium for fluorescence with DAPI (Vector). All samples were evaluated immediately after staining using an Axioskop 40 fluorescence microscope (Zeiss, Germany) at 460 nm for DAPI and 520 nm for TUNEL fluorescence and analyzed by Alpha digi doc 1201 software (Alpha Innotech, San Leandro, CA, USA).

### Statistical analysis

Statistical analysis was performed using commercially available software (SPSS 9.0 for Windows™). Differences in complement C5a levels in serum of *fB*-/- and *fB*+/+ mice were determined by the unpaired Student's *t*-test. A *P*-value < 0.05 was considered statistically significant.

## Abbreviations

C5a receptor (C5aR, CD88); central nervous system (CNS); cerebrospinal fluid (CSF); complement receptor type 1-related protein y chimeric molecule fused to mouse Ig (*Crry-Ig*); diaminobenzidine (DAB); 4',6'-diamino-2-phenylindole (DAPI); enyzme-linked immunosorbent assay (ELISA); factor B gene-deficient mice (*fB*-/-) and wild-type littermates (*fB*+/+); glial fibrillary acidic protein (GFAP); membrane attack complex (MAC, C5b-9); neuron-specific nuclear protein (NeuN); o-phenylenediamine dihydrochloride (OPD); phosphate-buffered saline (PBS); room temperature (RT); sodium dodecyl sulfate-polyacrylamide gel electrophoresis (SDS-PAGE); traumatic brain injury (TBI); terminal deoxynucleotidyl transferase (TdT); terminal deoxynucleotidyl transferase biotin-dUTP nick end labeling (TUNEL).

## Competing interests

The author(s) declare that they have no competing interests.

## Authors' contributions

IL, OIS, and PFS were responsible for conception and planning of the experiments, performing of all animal experiments, analysis of the data and writing of the manuscript. VMH and JMT provided the factor B knockout mice and wild-type littermates and assisted in the conception of the experiments and analysis of the data. IL and DH performed the TUNEL and immunohistochemistry experiments. MP and MET performed the Western blot experiments. DR, MHL, and PAW performed the murine C5a ELISA experiments and assisted in the interpretation of the results. WRS assisted in analysis of the data and writing of the manuscript.
